# Review of Zinc Oxide Piezoelectric Nanogenerators: Piezoelectric Properties, Composite Structures and Power Output

**DOI:** 10.3390/s23083859

**Published:** 2023-04-10

**Authors:** Neelesh Bhadwal, Ridha Ben Mrad, Kamran Behdinan

**Affiliations:** Department of Mechanical and Industrial Engineering, University of Toronto, Toronto, ON M5S 3G8, Canada

**Keywords:** zinc oxide nanostructures, piezoelectric nanogenerators, piezoelectric energy harvesting, piezoelectric thin films, power output, nanoscale piezoelectric, nanocomposite, structural design, energy density, lead free piezoelectric

## Abstract

Lead-containing piezoelectric materials typically show the highest energy conversion efficiencies, but due to their toxicity they will be limited in future applications. In their bulk form, the piezoelectric properties of lead-free piezoelectric materials are significantly lower than lead-containing materials. However, the piezoelectric properties of lead-free piezoelectric materials at the nano scale can be significantly larger than the bulk scale. This review looks at the suitability of ZnO nanostructures as candidate lead-free piezoelectric materials for use in piezoelectric nanogenerators (PENGs) based on their piezoelectric properties. Of the papers reviewed, Neodymium-doped ZnO nanorods (NRs) have a comparable piezoelectric strain constant to bulk lead-based piezoelectric materials and hence are good candidates for PENGs. Piezoelectric energy harvesters typically have low power outputs and an improvement in their power density is needed. This review systematically reviews the different composite structures of ZnO PENGs to determine the effect of composite structure on power output. State-of-the-art techniques to increase the power output of PENGs are presented. Of the PENGs reviewed, the highest power output belonged to a vertically aligned ZnO nanowire (NWs) PENG (1-3 nanowire composite) with a power output of 45.87 μW/cm^2^ under finger tapping. Future directions of research and challenges are discussed.

## 1. Introduction

To reduce greenhouse gas emissions and move towards sustainable energy, different energy conversion methods are being used to convert ambient energy into electrical energy via the piezoelectric, triboelectric, and pyroelectric effects, to name a few [1,2,3]. Several piezoelectric materials are being developed to harvest energy from ambient vibrations in the environment. Piezoelectric materials can convert mechanical energy (vibrations) into electrical energy due to the arrangement of dipoles in their structure. Compared with chemical batteries, they are environmentally friendly and can provide sustainable electrical energy [4]. Therefore, they are considered to have excellent potential for sustainable energy applications. Electronics in space, biomedical implants, and structural health monitoring (SHM) would greatly benefit from such a system. For example, several medical implants which are battery-powered must be removed and replaced from the patient via surgery due to the battery running low [5]. A piezoelectric energy harvester could harvest electrical energy from heartbeat vibrations and power or complement the battery in a pacemaker, prolonging the time before surgery for replacement is needed [5,6]. The use of piezoelectric energy harvesters for this application can help reduce infections, medical complications, and the overall cost of surgery Piezoelectric energy harvesters could power SHM sensors in hard-to-reach places, such as the underside of a bridge, by converting ambient vibrations from sources, such as cars passing over a bridge, into electrical energy to power the SHM sensor, thus reducing maintenance costs. For space flights, reducing the mass of the spacecraft increases mission capability and reduces cost [7]. Thus, lightweight piezoelectric energy harvesters that harvest ambient vibrations on the spacecraft and generate electricity are essential. Flexible lightweight weight piezoelectric energy harvesters can be integrated into fabrics to power wearable devices, such as microprocessors or LEDs, from the body motion of the user. 

Lead-containing piezoelectric materials typically show the highest energy conversion efficiencies; however, due to the toxicity of lead, as well as its harmful environmental impacts, lead-based materials will be limited in future applications [4]. Lead-free piezoelectric materials, such as barium titanate (BaTiO_3_), zinc oxide, and polyvinylidene fluoride (PVDF), are currently being developed for use in piezoelectric energy harvesters [4,6,8,9,10,11]. In bulk form, the piezoelectric properties of lead-free piezoelectric materials are significantly lower and can be an order of magnitude less than lead-containing materials. For example, the piezoelectric strain constant (d33) for traditional lead zirconate titanate (PZT) ceramics range from ~300 to 600 pm/V depending on the type of PZT [12,13], while bulk ZnO has a d33 of ~12.4 pm/V [14]. However, the piezoelectric properties of lead-free piezoelectric materials at the nano scale can be significantly larger than the bulk scale due to the enhanced role of surfaces, interfaces, reduced defects, stress concentrations, material variations, free boundaries for volume expansion/contraction, high crystallinity, and good control over the growth direction [6,8,15,16,17]. Recent advancements in the fields of manufacturing and nanotechnology have enabled the development of lead-free piezoelectric nanostructures, which are currently being modified and manipulated to have better energy conversion efficiency [4,6,8,15,18]. These nanostructures, when used for energy harvesting, are called piezoelectric nanogenerators (PENG). They are more flexible than their larger counterparts and show potential to be used for electrical energy harvesting. This review article will focus on ZnO nanostructures.

**Figure 1:**
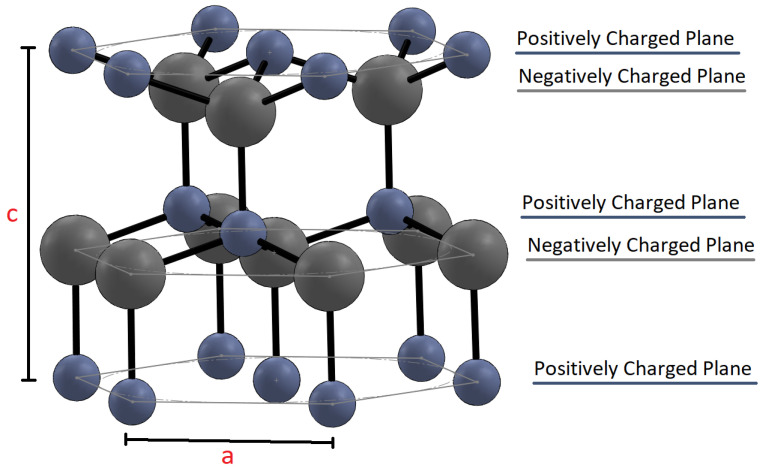
ZnO hexagonal crystal structure with oxygen shown in grey and zinc in blue. The lattice parameters a and c are shown.

ZnO is a piezoelectric material as well as a wide band gap semiconductor (3.37 eV) [14,19]. It has high transparency to visible light and is biocompatible and nontoxic [20,21,22,23,24]. It is present in a hexagonal structure (wurtzite), cubic structure (rocksalt), as well as another cubic structure (zinc blende), but the hexagonal structure is the most common, in which the Zn^2+^ cation is surrounded by four O^2−^ anions and vice versa [15,25]. ZnO in a wurtzite crystal structure is non-centrosymmetric due to the tetrahedral arrangement with lattice parameters a = 0.3296 and c = 0.52065 nm as shown in Figure 1 [15,20]. In the ZnO tetrahedron, the four bond lengths are not the same [25,26,27,28,29]. The length of the bond on the c- axis is longer than the other three bonds. As a result, the dipoles do not cancel out, leading to polarity along the axis of the unit cell [15,20,26,27]. When pressure is exerted on the corner of the tetrahedron, the centers of mass of the cations and anions are further displaced [20]. This results in a net polarization and piezoelectric behavior if all the tetrahedrons have mutual alignment. 

The structure of ZnO can be simply described as a series of alternating planes composed of tetrahedrally coordinated Zn^2+^ and O^2−^ ions, stacking alternatively along the *c*-axis and hence the crystal is terminated with a polar surface. Because of these polar surfaces, ZnO has higher surface energy on the (0001) and (000-1) surfaces when compared to other planes. This results in ZnO crystals having a higher growth velocity along the c-axis than other directions, which leads to the formation of 1D ZnO nanowires (NWs) [30]. Different morphologies are possible by blocking the growth of ZnO along the c-axis or by altering the kinetic parameters of different crystallographic planes [30]. In general, pure ZnO does not display ferroelectric behavior [8,31].

Current ZnO PENGs can power very-low-power-consuming electronics and their power density must be further developed to enable their use in more practical applications. Increased power density will also result in a reduction in PENG size in certain applications. To increase the power density of these harvesters, ZnO nanostructures with high piezoelectric properties should be used and the internal structure of the harvester, if in a composite form, should be optimized to maximize electrical power generation. This article reviews the suitability of Zinc Oxide (ZnO), a lead-free piezoelectric material, for use in piezoelectric energy harvesters based on the reported piezoelectric properties of the nanostructures. Then, the reported power outputs of different ZnO nanocomposite structures are compared and discussed. Although several review papers on ZnO energy harvesters exist, this is the first review that systematically reviews the different composite structures of ZnO PENGs from a power output maximization point of view [8,15,16]. The reported ZnO nanostructure with the highest piezoelectric properties and the ZnO PENG with the highest power output found in the review are discussed.

## 2. Effect of Purity and Morphology on the Piezoelectric Properties of ZnO

ZnO has and is being investigated for use in piezoelectric electric energy harvesters. The piezoelectric properties of lead-free piezoelectric materials at the nano scale can be significantly larger than the bulk scale [6,8,15,16,17]. The piezoelectric strain constant, d33, is the ratio of the strain produced to the applied electric field (m/V), or the ratio of electric charge generated per unit area to an applied stress (C/N) [32]. Bulk ZnO has a d33 of ~12 pm/V [14]. This is significantly lower than traditional lead zirconate titanate (PZT) ceramics, which have a d33 coefficient ranging from ~300 to 600 pm/V depending on the type of PZT [12,13]. 

**Figure 2:**
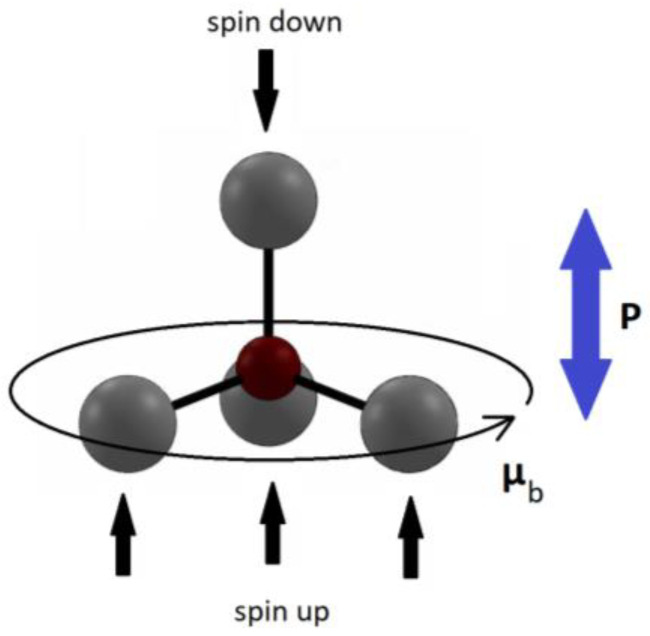
A repeating tetrahedral unit from the hexagonal ZnO structure with a zinc vacancy in red (where a zinc ion would have been) and oxygen ions in grey. The spin polarization leads to the induction of an electric dipole (P).

As shown in Table 1, the d33 values for pure ZnO nanostructures range from 0.4 to 80.8 pm/V, where Φ and L are the diameter and length of the NWs. Nanosheets (NSs) show the highest d33, followed by nanorods (NRs) and then nanobelts. Mahmood et al. synthesized ZnO NSs that were ~1.1 nm (2.5-unit cells) thick on a SiO_2_ substrate and recorded a d33 of 80.0 ± 0.8 pm/V [33]. The large covalent interaction with the substrate introduced high polarization and asymmetry in the ZnO structure, resulting in high piezoelectric performance [33]. Ghosh et al. observed a piezoelectric coefficient of ~49.7 pm/V in undoped ZnO NRs [34]. They found stacking faults in the wurtzite NRs (along the c-axis direction) due to zinc and oxygen vacancies. Ghosh et al. explained that the zinc vacancies led to the formation of magnetic moments due to the generation of upward and downward spin polarization in the nearest unpaired oxygen ions, which induced reversible electric dipoles and, hence, this resulted in a high piezoresponse (see Figure 2) [34]. Li et al. found that decreasing the size (diameter) of micropillars from 7 μm to 1.5 μm improved the piezoelectric coefficient from 18.2 to 46.9 pm/V [35]. This was explained to be due to an increase in polarization per unit volume as the diameters of the NWs decreased [35,36]. Zhao et al. characterized the performance of ZnO nanobelts and found a piezoelectric coefficient of 26.7 (pm/V) [37]. They suggested that the possible reason for the observed enhanced piezoelectric response is due to its perfect single crystallinity and freedom of dislocation [37]. 

When ZnO is subjected to a force it develops a voltage (piezo potential). However, due to its semi-conductor behavior the electrons in the material move towards the charges developed and diminish the total piezopotential, thus reducing the total energy harvested from the piezoelectric device [8,15,43]. This phenomenon is known as the internal screening effect. When piezoelectric potential is generated, some electrons also leak from the ZnO to the external circuit. Again, this effect reduces the total piezopotential generated. This phenomenon is known as the external screening effect [8,15]. It is important to note that ZnO is naturally an n-type semiconductor due to intrinsic defects from the methods of preparation [8,15]. A common method to decrease the screening effect is to p-type dope the ZnO to reduce the excess free electrons in the material [16]. Doping can also cause strain to the ZnO structure, which can further enhance the polarity and piezoelectric properties and induce ferroelectricity, see Table 2 [16]. Doping concentration and chemical inhomogeneity can affect the piezoelectric properties, which need to be carefully controlled [44,45,46]. 

Sinha et al. concluded that dopant ions that improve the piezoelectric performance of ZnO have a higher ionic charge to ionic radius ratio (ionic charge)/(crystal or ionic radius) than the host ion Zn^2+^ (2.70 e/Å) [47]. This is because there is larger polarity in the dopant-O bonds and easier bending of bonds along the polar c-axis under applied electric field, leading to larger piezoelectric coefficients [47]. Table 2 shows the d33 values for different doped ZnO nanostructures.

Laurenti et al. studied vanadium-doped ZnO films. After rapid thermal annealing, better c-axis-oriented V-doped ZnO thin films were obtained and there was a partial conversion of the starting V^+3^ ions into V^+5^ [46]. The stronger polarity of both V^+3^-O and V^+5^-O chemical bonds, together with the corresponding easier rotation under the application of an external electric field, resulted in a d33 of 85 pm/V [46].

Sinha et al. synthesized Y-doped ZnO NSs with a d33 of 420 pm/V [47]. It was explained that the polar area of the (001) plane in sheet morphology is larger than that for rod morphology, hence the large d33 [47]. Y also has an ionic radius ratio (ionic charge)/(crystal/ionic radius) of 2.88 e/Å, which is larger than that of zinc [47]. Hence, the Y-O bonds possess stronger polarity than Zn-O bonds, which makes the rotation of Y-O bonds along the c-axis easier [47].

Batra et al. studied Nd^3+^-doped ZnO NRs (note the ionic charge to radius ratio of Nd^3+^ dopant ion is 3.05 e/Å, which is greater than that of host Zn^2+^ ions 2.70 e/Å) [48]. Doping significantly enhanced the d33 value of ZnO, with the Nd-doped ZnO having a d33 value of 512 pm/V which is comparable to PZT in bulk form and shows the potential of ZnO as a piezoelectric material candidate for PENG.

**Table 2:** d33 Values for Doped ZnO Structures.

	d33 pm/V or	Purity/Form	Dimensions	Measurement Method	
ZnO bulk	12.4	-	-	-	[14]
NRs	43.38	Eu^+3^ doped	Φ 78.92 nm L = 302.33 nm	D-V Curve	[49]
	45.49	Gd^+3^ doped	Φ 59.85 nm L = 245.13 nm	D-V Curve	[50]
	100.30	La^+3^ doped	Φ 123.49 nm L = 704.38 nm	D-V Curve	[51]
	30	Ce^+3^ doped	Φ 16–20 nm L = 80–120 nm	D-V Curve	[52]
	512	Nd doped	Φ 101 nm L = 412 nm	D-V Curve	[48]
	41.28	Ba^+2^ doped	Φ 73.25 nm L = 476.51 nm	D-V Curve	[53]
NSs	420	Y^+3^ doped	~34 nm	D-V Curve	[47]
Film	85	V-doped (Si substrate)	285 nm thick	D-V Curve/ AFM	[46]

Several studies found that the piezoelectric output of ZnO NWs is negatively affected by humidity, and researchers should account for this while manufacturing and testing ZnO nanostructures and PENGs [54,55,56].

## 3. Piezoelectric Energy Harvester Structure and Power Output

The following sections focus on the power output and design of different ZnO PENG composites. The most used method for synthesizing ZnO nanostructures is presented first followed by methods for addressing the screening effects that lower the effectiveness of energy harvesters. 

### 3.1. Synthesis and Hydrothermal Growth of ZnO Nanostrucutres

The hydrothermal method consists of the deposition of a seed layer of ZnO onto a substrate, typically by sputtering, spin coating, or dip coating, followed by the growth of nanostructures in a zinc-rich solution at temperatures that are typically less than 100 °C. 

After seed layer deposition on the substrate, it is placed into an autoclave that is filled with a heated solution, typically comprising zinc nitrate hexahydrate and hexamethylenetetramine (HMTA) in deionized (DI) water. The elevated solution temperature causes the thermal decomposition of zinc nitrate hexahydrate, which provides the required Zn^2+^ ions, and HMTA, which provides hydroxyl ions [57]. This leads to the formation of Zn(OH)_2_ and then ZnO [57,58]. These ions attach on the seed layer to form ZnO nanostructures. If the temperature is too high, the additional energy can cause the homogeneous precipitation of ZnO in the solution [57].

Several factors of the growth solution affect the final nanostructure:

1. The hotter the temperature of the solution, the faster the growth rate of the nanostructures. Different temperatures can also lead to different morphologies, such as nanoballs (below 75 °C) and NRs (above 95 °C) [57,59].
2. Acidic medium is not favorable for the growth of ZnO NRs. The ideal solution pH lies between 7 and 10 [60]. The average length and diameter of NRs enlarge with the increase in the pH of the solution.
3. Replacement of the growth solution with a fresh solution can increase the nanostructure growth rate, height, and diameter/thickness (slightly) due to the replenishment of depleted ions [57].
4. Seed layer density and orientation: Growth of ZnO along the c-axis is energetically favorable and thus the orientation of the c-axis in the seed layer dictates the direction of growth of nanostructures in the PENG [57]. Larger grain sizes in the ZnO seed layer, which are typical in thicker films, result in thicker nanostructures. When ZnO seed nanoparticles are deposited via dip coating or spin coating, the number of seeds is lower than in thin-film seed layers and the density of seeds can be increased by repeating the process multiple times and/or increasing the concentration of solution. Increasing the density of nanostructures results in the structures being more perpendicular to the substrate as the dense nanostructures only allow its neighboring structures to grow similarly [61]. Low density results in the nanostructures being able to grow freely and with less overall alignment.

To synthesize doped ZnO nanostructures prepared by the hydrothermal method, often a doping reagent is added during hydrothermal growth, for example lanthanum nitrate hexahydrate (La(NO_3_)_3_⋅6H_2_O) for lanthanum doping and neodymium nitrate hexahydrate (Nd(NO_3_)_3_·6H_2_O) for Nd^3+^ doping to name a few [48,62].

Note that a longer growing duration leads to longer ZnO nanostructures. Additionally, the abundant oxygen sources in aqueous solution generate oxygen vacancies promoting n-type semiconductor behavior in ZnO [15].

### 3.2. Methods for Dealing with Screening Effects

Common techniques used across different composite structures to decrease the screening effect of ZnO are the following [15]:

(1) **Doping**: P-type doping reduces excess free electrons by replacing the majority carriers (electrons) to holes as well as distorting the crystal lattice, increasing polarity [8,16]. Note that n-type doping increases lattice straining along the ZnO polar axis but does not reduce the electron screening effect [8].

(2) **Surface Treatment**: Annealing removes absorbed oxygen ions as well as induces crystallinity [8]. Surface passivation can include coating/encapsulating the surfaces of the nanostructures to reduce surface electrons from ejecting into the bulk material [8]. The coating/encapsulation also prevents short circuiting of neighboring nanostructures during the current measurement.

(3) **Interfacial modification**: In this technique, a p-n junction or Schottky contact between ZnO and the electrode is created by placing a thin insulating layer between them [8,59,63]. The p-n junction depletes the excess electrons in the ZnO, reducing the internal screening effect. Additionally, the electrode/ZnO Schottky barrier reduces leakage current through the interface (external screening effect). 

Piezoelectric composites are defined by the number of dimensions through which the material is continuous (for example 1-3, 0-3, etc.) [64]. By convention, the first digit refers to the piezoelectrically active phase. There can be multiple active phases. 

### 3.3. Vertically Aligned ZnO NWs PENG (1-3 NW Composites)

**Figure 3:**
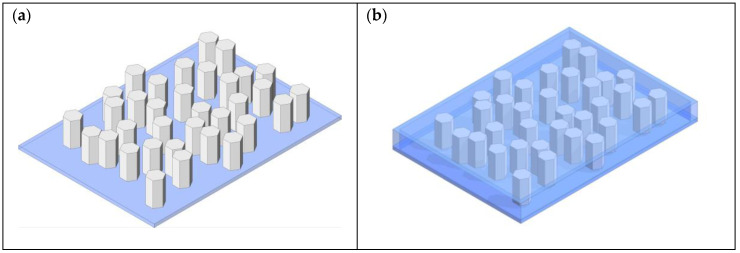
Vertically aligned ZnO NW PENG (1-3 NW composite). ZnO NWs are depicted by the hexagonal cross-section pillars and typically do not have equal spacing between them. (**a**) The NWs left in air grown on a substrate; (**b**) the encapsulated NWs, depicted by the blue matrix.

An aligned ZnO NW composite (e.g., 1-3 composites) has ZnO NWs continuous in one direction, while a second material (could be piezoelectrically active or passive) is continuous in all three directions as shown in Figure 3. In Figure 3, the ZnO NWs are continuous in the vertical direction and encapsulated in either a second material or left in air, which is continuous in all three directions (vertical and both horizontal directions), hence forming a 1-3 composite. Note that the NWs have hexagonal cross sections and are not evenly spaced. Typically, it is desirable to manufacture this type of composite such that the c-axis of the ZnO crystals is parallel to the length of the NWs and perpendicular to the substrate. The hydrothermal method is the most used method of synthesizing NWs in 1-3 composites. After the NWs are formed on the substrate which usually serves as the first electrode, they are then either encapsulated or left in air, after which a second electrode is applied to the top of the NWs. 

Of the structures discussed in this review, 1-3 composites show the highest power outputs due to good alignment and higher piezoelectricity of NWs (piezoelectricity increases with aspect ratio [65]) and crystallinity. Table 3 shows the output power, voltage, and current of several 1-3 ZnO composites. In the table, power density, force, and resistor are the electrical power produced by the PENG per unit area, loading conditions on the PENG during the power measurement, and the value of the external resistor connected to the PENG during power measurements.

The generation of DC was studied where researchers developed a 1-3 ZnO nanocomposite which had two 1-3 composites stacked on top of each other and comprised a copper layer (substrate), ZnO NWs, polyacrylonitrile (PAN) film, ZnO NWs, and then a gold electrode [66]. Note that PAN is a piezoelectric material. The gold layer formed a Schottky contact with the ZnO NWs and the copper layer formed an ohmic contact, which led to a rectified DC output from the PENG without the use of a rectifier bridge as well as a reduced screening effect at the gold-ZnO interface. The addition of a PAN layer and double ZnO NWs increased the output voltage and current more than 5 and 2.3 times, respectively. 

Ou et al. used a porous polycarbonate template (Whatman filter paper) to control the alignment of the ZnO NWs [67]. Polycrystallinity due to the ZnO nanoparticles fusing together was observed in the NWs after growth in the template and the authors expect higher piezoelectric performance if the c-axis of the crystals were oriented parallel to the length of the nanowire. The PENG was fatigue-tested over a period of 4 months with negligible degradation in performance.

Development of 1-3 composites on flexible textile substrates is pursued for applications that require significant flexibility. Zhang et al. grew ZnO NWs on a silver-paste-coated nylon fabric via a novel hydrothermal method to form a composite similar to Figure 3 [68]. Schottky contact between ZnO NRs and Ag electrodes helped reduce the screening effect. The ZnO NWs had a high length/diameter ratio (aspect ratio) of 40:1, which enhanced the NWs’ piezoelectric behavior. The values of output voltage were stable after 1000 cycles of testing. In another study, ZnO NWs were grown on a copper (Cu)/nickel (Ni)-coated polyester textile [69]. Copper(I) thiocyanate (CuSCN) was coated on ZnO NWs via drop coating to passivate the surface of the wires via the formation of p-n junctions, which reduced the screening effect. This was followed by the deposition of a layer of Poly(3,4-ethylenedioxythiophene) poly(styrenesulfonate) (PEDOT: PSS) (an organic semiconducting polymer) on top of the CuSCN/ZnO NWs via spray coating to form a p-n junction with the surface of the NWs and decrease the screening effect. The PENG was encapsulated in polydimethylsiloxane (PDMS) and demonstrated stability over ~26,000 cycles of testing. The researchers found that by increasing the length of ZnO NWs, the PENG exhibited increasing power density and they expected that longer NRs would have more strain when subjected to the same vibrations [69].

The addition of secondary piezoelectric materials into the composite can increase power generation. Anand and Bhatnagar developed a 1-3 ZnO composite and passivated the surface of ZnO NWs with PVDF, which is also piezoelectric [70]. The vertically aligned ZnO NRs played a pivotal role in the nucleation, stabilization, and increase in the amount of β phase in the PVDF, which is the phase that shows the strongest piezoelectric properties. The PENG without any poling produced a power output which was 1800 times more than that of just PVDF.

One study developed a double-junction epitaxial double heterostructure, i.e., ZnO NWs were grown on both sides of a single layer of graphene [71]. The total output voltage and current density were improved two-fold compared to an epitaxial single heterostructure.

Several studies have focused on decreasing the screening effect to increase the power output. Lu et al. studied the effect of passivating ZnO NWs via depositing gold nanoparticles on the surface of the NWs via photothermal deposition [72]. The Schottky junctions formed at the gold-ZnO interface led to a decrease in the screening effect. A 1~2 μm layer of poly(methylmethacrylate) (PMMA) was deposited on the top of the NWs to weaken the screening effect. The output voltage and current density of the gold nanoparticles ZnO 1-3 composite was 10 times higher than the output of pristine ZnO nanoarray-based piezoelectric nanogenerators. Dahiya et al. studied different encapsulation materials for passivating the surface of ZnO NWs in a 1-3 composite [73]. Parylene C deposited by chemical vapor deposition (CVD) was found to be the best encapsulant considering its effectiveness of infiltration between the ZnO NWs, and its conformity, thickness controllability, and compatibility with flexible substrates. In one study, ZnO p-n homojunction NWs were grown with lithium-doped segments directly on top of intrinsic NWs (n-type) encapsulated in PMMA [63]. The open-circuit voltage was enhanced 4.5-fold by controlling the doping concentration via a reduction in the screening effect. The addition of a layer of MoO_3_ (5–10 nm thick) between the top of the NWs and the electrode reduced the leakage current and substantially improved the piezoelectric NG open-circuit voltage 4.3-fold. In one study, a 1-3 composite with La-doped ZnO NWs was synthesized and showed a 1.4 times increased output voltage performance attributed to the decrease in the electron screening effect [62].

**Table 3:** Power, voltage, and current output for 1-3 composite nanowires (note: PEMs, OC, and SC stand for piezoelectric materials, open circuit, and short circuit, respectively).

	PEMs	NW Dimensions	Preparation Methods	V (V)	I (μA)	Resistor (MΩ)	Power Density (μW/cm^2^)	Force
[66]	ZnO/PAN	Ø 140 ± 50 nm Length 3 ± 0.2 μm	Hydrothermal	1.6	16.4	0.45	5.86 Peak	10 N, 1 Hz
[67]	ZnO	Ø ~227 ± 92 nm, Length ~12 μm	Hydrothermal	-	-	1	0.0019 Peak	75 Hz and amplitude of ~1 mm
[68]	ZnO	Ø 100 nm Length ~4 μm	Hydrothermal	4	0.020	-	-	Palm clapping
[69]	ZnO	Length ~5.9 µm (Thickness of CuSCN is ~2.5 nm)	Hydrothermal + Drop coating + Spray coat	1.32 OC	0.56 SC	2-7	0.67 Average	26 Hz on a resonant cantilever
[72]	ZnO	Ø ~200 nm (Au NPs with Ø of several tens of nm)	Hydrothermal + Photothermal Deposition	2 OC	1/cm^2^ SC	0.0917 internal impedance	-	Finger force
[70]	ZnO/PVDF	Ø 220 nm	Hydrothermal + Drop Cast	46.64	1.392 SC	15	45.87	Finger Tapping ~12–14 kPa
[73]	ZnO	Ø 50 nm ± 20 nm Length 0.5 µm ± 0.1 µm	Hydrothermal + CVD	-	-	20	4.38 Peak	Hand Tapping 100 kPa
[71]	ZnO	Ø 60 to 100 nm Length ~2.50 μm	Hydrothermal	0.17	0.0275/cm^2^	-	0.004 Peak	Compression 49 N
[63]	Li doped ZnO	Ø 100 nm–200 nm Length ~4 μm	Electrochemical	1.95 OC	0.075	-	-	5 Hz and 3.5 m/s^2^
[62]	La doped ZnO	Ø 176 nm Length ~1.6 μm	Hydrothermal	3	-	-	-	-

### 3.4. Vertically Aligned ZnO 2D Composite (1-3 2D Composites)

**Figure 4:**
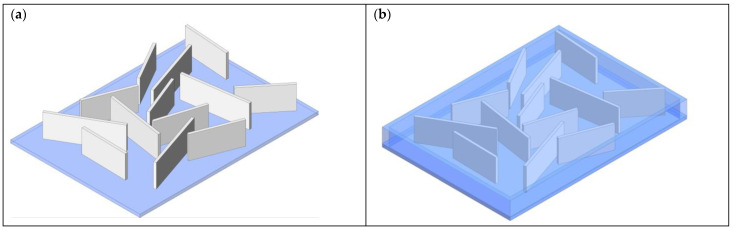
Vertically aligned ZnO 2D PENG (1-3 2D composite). ZnO 2D structures are depicted by the grey sheets. (**a**) The 2D nanostructures left in air grown on a substrate; (**b**) the encapsulated NSs depicted by the blue matrix.

Like 1-3 NWs ZnO composites, 1-3 ZnO composites with 2D nanostructures have ZnO continuous in one direction while a second material (could be either piezoelectrically active or passive) is continuous in all three directions. However, instead of ZnO being present in the form of NWs, they are present in the form of 2D geometries, such as NSs and nano discs (NDs), as shown in Figure 4. Generally, ZnO 1-3 2D composites contain ZnO NSs with their length perpendicular to the substrate encapsulated in a medium. Again, typically, it is desirable to manufacture this type of composite such that the c-axis of the ZnO crystals is perpendicular to the substrate. The 1-3 ZnO composites with 2D nanostructures are mostly grown via the hydrothermal method. After the aligned nanostructure is formed on the substrate (which usually serves as the first electrode), it is then either encapsulated or the nanostructures are left in air and then the second electrode is applied.

ZnO NSs have

1. Better mechanical durability when compared to NWs [65].
2. Lower piezoelectric properties than 1D nanostructures as the piezoelectric coefficient decreases with decrease in aspect ratio [65]. Output voltage increases with increase in aspect ratio [74].
3. Lower electron diffusion coefficient (D_n_) than NWs, i.e., lower screening effect [59,75].
4. A growth mechanism in which a dopant attaches to the ZnO on top of the c-axis during hydrothermal growth. This limits the growth of ZnO along the c-axis and promotes the growth of NSs instead of NWs. For example, the dopant can be aluminum cation from an aluminum substrate that the ZnO nanostructures are grown on [59,76].

Although the highest power output reviewed is a vertically aligned ZnO NW composite (1-3 NW ZnO composite) [70], several studies on vertically aligned ZnO 2D composites (1-3 2D composites) synthesized both 1-3 2D composites as well as 1-3 NW ZnO composites for comparison and found that the output of the 1-3 2D composites was higher [59,77].

Table 4 shows the output power, voltage, and current. Mahmud et al. developed a 1-3 ZnO composite with both 1D NWs and 2D NSs with the aim of making the harvester more robust in terms of load handling [59]. Aluminum cations from the substrate limited growth along the c-axis and led to the formation of NSs and the top half of the nanostructures were doped with Li to form a homojunction. PMMA encapsulated the structures as well as formed a thin layer on top of the nanostructures to form a semiconductor–insulator–metal junction with the electrode, which reduced the external screening effect. The average peak-to-peak output open-circuit voltages were 1.35 times higher for the PENG with both the NWs and NSs as compared to a PENG grown in similar conditions with only 1D NWs, and this was attributed the integration of 1D/2D ZnO nanostructures.

Multiple studies looked at decreasing the screening effect. Gupta et al. synthesized vanadium-doped ZnO NSs in a 1-3 composite in a similar structure to Figure 4, which generated a direct current [78]. The output current from the V-doped ZnO NS-based NG was ~100 times more than an undoped ZnO NW-based PENG prepared the same way due to a reduction in the screening effect and the network structure of the NS. When force was applied along the vertical direction, the stress was distributed through the network to all NSs under the force-applied area, which greatly enhanced the NG’s performance. The PENG generated a DC-type current due the top side of the V-doped ZnO NS being negatively charged due to [V(OH)_4_^−^] being present from hydrothermal growth, which did not allow the back flow of accumulated electrons from the bottom electrode to the top electrode due to electrostatic repulsive force. In another study, Br-doped ZnO NSs were synthesized in a 1-3 composite form with a similar structure to Figure 4 [79]. The NSs were encapsulated in PDMS and the aluminum oxide on the substrate acted as an insulating layer by generating a potential barrier between ZnO and the bottom electrode, which restricted the recombination of charge carriers. The current and voltage output of the Br-doped ZnO PENG was around three times that of undoped ZnO NSs PENG because Br doping reduced the screening effect and improved the piezoelectricity of NSs.

In one study, a hybrid piezoelectric and triboelectric nanogenerator was developed where the piezoelectric component 1-3 composite comprised vertically aligned NSs [61]. The study found that the ZnO NSs grown along the c-axis orientation exhibited the best piezoelectric response.

In one study, 2D-like ZnO nano discs were synthesized in 1-3 composite form. ZnO seed layers were deposited by spin coating on the substrate followed by annealing at 150 °C ten times, leading to the formation of a dense ZnO seed growth layer [80]. It was proposed that due to the density of the seed layer and the surplus OH- ions, which block growth along the c-axis during hydrothermal growth, nano discs were formed. The PENG generated a DC-type electric signal which may have been due to the presence of OH- ions at the top of the nanodiscs via a mechanism similar to the one shown by Gupta [78]. After fabrication, the entire PENG was annealed at 150 °C for 2 h, which lead to an approximately eight-fold voltage increase. 

In one study, a two-step growth method of ZnO NSs (NSs) was explored to form a similar structure to Figure 4 [77]. NWs were grown via the hydrothermal method. For the second step of the growing, the NWs were placed into a solution rich in NaOH, which led to a high concentration of OH- in the precursor solution which then bonded to the c-axis of the ZnO, completely changing the NWs into NSs. The PENG demonstrated a four-times better direct current output than those based on NWs. It was also found that the output current increased as the 2D nanostructures became thinner.

Several studies focus on generating direct current via ZnO NSs in 1-3 composites, where the ZnO NSs are grown on top of layered double hydroxides (LDH) [65]. When NSs or NWs bend due to force applied perpendicular to the NS, one side of the sheet undergoes compression while the other side undergoes tension, which leads to piezopotential being generated across the thickness of the sheet [81]. If one of the two electrodes forms a Schottky contact with the ZnO, the electrons in the NSs will only be able to flow in one direction and thus direct current is observed. Tilted ZnO nanostructures in a 1-3 composite are more likely to bend and hence generate DC. As NSs are more mechanically stable than NRs they are preferred for DC generation.

**Figure 5:**
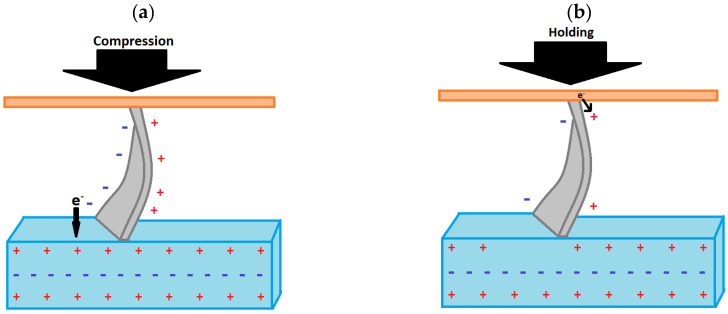
(**a**) Mechanism of charge transfer in LDH under compression; (**b**) mechanism of charge transfer in the LDH under holding.

Kim et al. developed 2D ZnO NSs/ZnAl-LDH networks on an aluminum substrate via hydrothermal growth [65]. Gold-coated polyether sulphone (PES) was used as a top electrode and formed a Schottky contact with the top of the ZnO NSs. It was proposed that when compressed, negative charge on the nanosheet moved to the positively charged layer of the LDH that interfaced with the NSs, as shown in Figure 5. As a result, the LDH layer had net negative potential. Then, the positive potential in the ZnO NSs attracted electrons from the top electrode to overcome the Schottky barrier between the top electrode and the ZnO NSs and reduced the piezoelectric potential. This process generated DC. 

Lee et al. also grew ZnO NSs and Zn-LDH between ZnO NSs and Al substrates [76]. A Au-coated polyethylene naphthalate (PEN) substrate was placed above the ZnO nanosheet network as a top electrode and formed a Schottky contact. The hydrothermal growth solution concentration was found to affect the tilting and density of the NSs. As the ZnO NSs needed to bend to exhibit strong DC power generation, tilted NSs were grown. The PENG showed stability for over 4000 cycles and generated DC. In one study, ZnO NSs were grown on both sides of an aluminum substrate to enhance the power output of the PENG [82]. The existence of a ZnAl: LDH layer at the interface of ZnO NSs and aluminum was confirmed, and the output was DC. The nanogenerator exhibited a ~1.7 times larger output voltage than that of a single-side-coated one.

**Table 4:** Power, Voltage and Current Output for 1-3 Composite Nanosheets.

No.	PEMS	Form	Preparation Methods	V (V)	I (μA)	Resistor (MΩ)	Power Density (μW/cm^2^)	Force
[59]	Li-doped ZnO	NWs Ø 50–120 nm 2D buckled NS ~50 nm thick ~1.3 μm long	Hydrothermal growth	10.18 OC	15.9 SC	10	8.4 Peak	5 N, 5 Hz
[78]	V-doped ZnO	NSs 900 nm–1.0 μm thickness of 15–20 nm	Hydrothermal + Spin Coat	-	1.0/cm^2^	-	-	Compressive force of 0.5 kgf
[79]	Br-doped ZnO	NS 15–31 nm thick	Hydrothermal	8.82	4.41	2	38.8962 Peak	Force of 3 kgf
[61]	ZnO	NSs Thickness ≤ 10 nm	Hydrothermal	130 OC	37 SC	-	-	7 N at 10 Hz
[80]	ZnO	Nanodiscs thickness ~30 nm Ø of 1.0–1.5 µm	Hydrothermal + Annealing	17	0.150/cm^2^	-	2.55	Compressive force 0.1 kgf
[65]	ZnO	NSs 80 nm and 3 μm	Hydrothermal	~0.75	~15/cm^2^	-	11.8	Push force of 4 kgf
[76]	ZnO	NSs 2.7 μm ± 0.8	Hydrothermal	0.9 OC	16.5/cm^2^ SC	0.010	0.6	4 kgf, 1 Hz
[82]	ZnO	NSs -	Hydrothermal	0.285 OC	-	~0.300	~0.011 Peak	Tapping force ~8–8.5 N
[77]	ZnO	NSs 0.5–1 μm thickness 60–80 nm	Two-step Hydrothermal		0.15	-	-	Compressive force of 1 kgf

### 3.5. Non-Vertically Aligned ZnO Composites (0-3 Composites)

**Figure 6:**
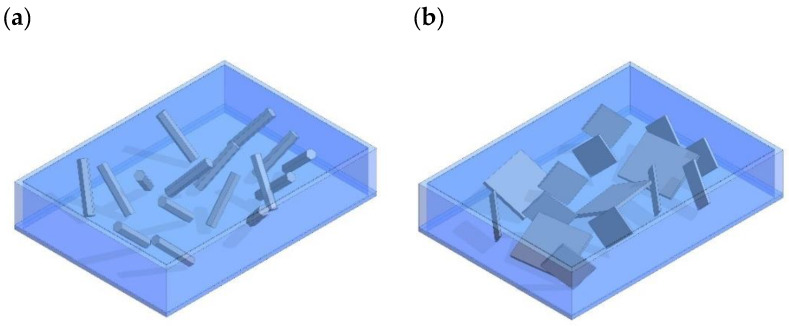
(**a**) 0-3 composites with ZnO NWs on the left; (**b**) 0-3 composites with ZnO NSs. The blue matrix represents the material the ZnO nanostructures are encapsulated in.

The 0-3 ZnO composites have noncontinuous ZnO nanostructures in all directions and are encapsulated in a second material (could be piezoelectrically active or passive) continuous in all three directions, as shown in Figure 6. A 0-3 composite with ZnO NWs is shown on the left while a 0-3 composite with NSs is shown on the left. The hydrothermal method is again the most used method for synthesizing ZnO nanostructures for use in 0-3 composites. In this case, no substrate or seed layer is placed in the hydrothermal growth solution and instead ZnO nanostructures are precipitated in the solution. The precipitate is then separated, cleaned, and mixed with an encapsulating material. This mixture is applied to a substrate via spin coating or drop coating followed by electrode placement, forming a 0-3 composite. Most studies use NRs in 0-3 composites while fewer use NSs.

The 0-3 composites are simpler to produce when compared to 1-3 composites but show lower power outputs due to the c-axis of the individual ZnO nanostructures being randomly aligned. Poling is used to increase alignment. Table 5 shows the output power, voltage, and current of several 0-3 ZnO composites.

In one study, a 0-3 composite with annealed (550 °C) lithium-doped NRs encapsulated in PDMS was synthesized [83]. The Li-doped ZnO NRs were ferroelectric and poled to enhance dipole alignment, which resulted in the output voltage increasing more than five times (poled at 105 kV cm^−1^ at 65 °C for 20 h). Note that the poling process had no impact on the piezoelectricity of the undoped ZnO NRs. The output voltage and current were stable during 1350 cycles every week for 4 weeks. 

Studies have focused on decreasing the screening effect. In another study, lanthanum (La)-doped ZnO NRs were encapsulated in PDMS to form a 0-3 composite [84]. An undoped PENG produced an output of around 5 V. The La-doped PENG produced an output of 18 V. Doping with La resulted in stronger polarity of La–O bonds and a reduction in the screening effect. Annealing of La-doped ZnO NRs further increased the device output close to 23 V. The device stability was tested over 4500 cycles and found to be extremely stable. Manikandan et al. investigated the piezoelectric response of Sn-doped ZnO NRs in a 0-3 composite with biodegradable paper substrate [85]. The Sn-doped PENG generated a voltage three times larger than a pristine PENG due to a reduction in the screening effect. Batra et al., who synthesized Nd-doped ZnO NRs with a d33 coefficient of 512 pm/V, developed a 0-3 composite using the Nd-doped ZnO NRs (heated at 500 °C for 6 h) mixed with PDMS [48]. The average value of open-circuit voltage from the Nd-ZnO NRs was ~15.5 times higher than pristine ZnO NR under finger-tapping mode without any electrical poling of the devices. The reduced screening effect and large *d*_33_ value from Nd doping resulted in enhanced performance.

Many studies add multiple piezoelectric materials into the composite. Anand and Bhatnagar, who developed a 1-3 ZnO NW-PVDF composite, also developed a 0-3 ZnO NR encapsulated in a PVDF composite [70]. The ZnO in the PVDF promoted β-phase growth and the relative amounts of β-phase in the nanocomposites were 53, 63.6, and 80.6% for pure PVDF, the 0-3 composite, and the 1-3 composite, respectively. The power density of the 0-3 composite was 30 times larger than pure PVDF but 59 times smaller than the 1-3 composite. Overall, this study found that 1-3 composites performed better than 0-3 composites under finger tapping. Sadaf et al. developed a 0-3 composite with Nd-doped ZnO along with multiwall carbon nanotubes (MWCNTs) incorporated in a PVDF polymer matrix [86]. The use of Nd-doped ZnO and MWCNT resulted in an organized crystalline structure with the γ phase and β phase being dominant in the composite. In one study, a Li-doped ZnO NR coated in polyethylene glycol (PEG) along with MWCNTs were incorporated in a PVDF matrix to form a 0-3 composite [87]. The PEG coating helped promote the ß phase of PVDF due to interactions between the O-H group of PEG and the H-C group of PVDF, which reduced the need for poling to increase the ß phase and power output.

Morphologies other than ZnO NRs have been used in 0-3 composites. Sinha et al., who synthesized Y-doped ZnO NSs with a d33 coefficient of 420 pm/V, developed a 0-3 composite using the Y-doped ZnO NSs encapsulated in PDMS [47]. The Y-doped ZnO composite showed 10 times higher output voltage compared to a pure ZnO NRs composite. The reduced screening effect, larger polar area (note that the c-axis is along the thickness of the sheet or the thinnest dimension), and large *d*_33_ value resulted in enhanced performance. In one study, V-doped ZnO NSs agglomerated in a flower-like morphology were synthesized and incorporated into a 0-3 composite [88]. The V-doped ZnO NSs exhibited ferroelectricity and were poled at 80 kV cm^−1^, which resulted in the output voltage and current increasing ~20 times due to a decrease in the screening effect. The reliability of the composite was measured over 1000 cycles per week for a month and showed stability.

**Table 5:** Power, Voltage, and Current Output for 0-3 Composites.

	PEMS	ZnO Nano Structures	Preparation Methods	V (V)	I (μA)	Resistor (MΩ)	Power Density (μW/cm^2^)	Force
[83]	Li-doped ZnO	NRs Ø 600–800 nm	Hydrothermal + Spin Coating + Poling	9	2.5	-	5.62 Peak	Bending induced by horizontal travel (5 mm) of a stage
[84]	La-doped ZnO	NRs Ø 65 nm	Hydrothermal +Spin coating + Annealing	5	-	100	5 Peak	2N compressive force
[85]	Sn-doped ZnO	NRs Ø 185 to 250 nm	Hydrothermal + Drop Casting	4.15 OC	0.036 SC	-	0.023	22 N
[70]	ZnO-PVDF	NRs Ø 114 nm	Hydrothermal + Drop Cast	-	-	-	0.77	Finger tapping 12–14 kPa pressure
[48]	Nd-doped ZnO	NRs Ø ~101 nm Length ~412 nm	Chemical Co-precipitation + Spin coat	31 OC	-	-	-	Finger tapping
[86]	Nd-doped ZnO/PVDF/ MWCNT	NRs Ø 20–70 nm	Chemical Co-precipitation + Drop Cast	75.8 OC	28.8 SC	1	12.55 Peak	Finger Tapping
[87]	Li-doped ZnO/PVDF/ MWCNT	NRs	Hydrothermal + Drop coating	8.1 OC	4/ cm^2^	-	-	Finger Tapping
[47]	Y-doped ZnO	NSs ~34 nm thick	Chemical Co-precipitation + Spin coat	20 OC	-	-	-	Finger tapping
[88]	V-doped ZnO	NSs forming Nanoflowers	Hydrothermal + Spin coating	32	6.2	-	-	Bending induced by horizontal travel distance of 10 mm

### 3.6. ZnO as an Additive to Electrospun Fibers

Although there are studies on ZnO electrospun fibers [89], most studies focus on ZnO as an additive for increasing the piezoelectric performance of other piezoelectric electrospun materials, such as PVDF and PAN. 

**Figure 7:**
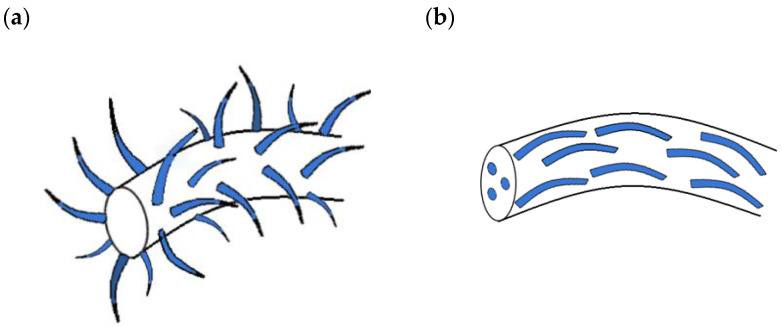
(**a**) ZnO NWs (depicted in blue) grown on the surface of another piezoelectric fiber (depicted in white); (**b**) ZnO NRs (depicted in blue) in a piezoelectric fiber of another piezoelectric material (depicted in white).

The growth of ZnO nanostructures on PVDF/PAN can increase the amount of *β* phase and zigzag phase, respectively, due to the nanoforces induced on the fiber surface during ZnO nanostructure growth, as seen in Figure 7a. An addition of ZnO nanostructures in the piezoelectric fiber can increase the crystallinity and preferable phase in the fiber due to interactions between the ZnO and piezoelectric fiber, as shown in Figure 7b. The increase in the preferable phase as well as the increase in the total amount of piezoelectric material results in an increase in piezoelectric performance in the PENG. Table 6 shows the output power, voltage, and current of several 0-3 ZnO composites.

The growth of ZnO NRs on the electrospun fiber has been studied by Sun et al., who grew ZnO nanorods on electro-sprayed PVDF fibers [90]. Tested under the same condition, the PVDF-ZnO PENG generated almost three times higher output voltage than a pure PVDF PENG prepared with the same method. The growth of ZnO NRs increased the *β* phase of PVDF from 70.8 to 73.2%, and it was proposed that the ZnO nanorods would deflect and slide against each other when vibrated, resulting in more deformation of ZnO NRs and PVDF fibers and the separation of ionic charges, thus increasing power output. In one study, ZnO NRs were grown on electrospun PAN fibers [91]. The ZnO successfully enhanced the planar zigzag conformation of the PAN nanofibers, and the PENG showed a 2.7-times improvement in piezoelectric performance compared to a PENG prepared without ZnO. 

Several studies add ZnO nanostructures into the electrospun fiber. In one study, ZnO nanoparticles (NPs) and NRs were compared as fillers to synthesize electrospun PVDF mats [92]. The NRs as fillers outperformed the NPs because the NRs aligned with the fiber axis due to the sink-like flow during electro spraying while the ZnO NPs were randomly oriented. The ZnO NRs also resulted in a higher (β) phase than ZnO NPs and the ZnO NRs with large aspect ratios were more easily deformed by external force. The output voltage of the NR-PVDF mat was ~1.4 times that of the NP-PVDF mat. In another study, the addition of ZnO NRs fillers increased the β-phase content and crystallinity in P(VDF-TrFE), which caused the PENG voltage to increase by approximately 500% compared to a PENG without ZnO NRs [93]. 

The reduction in the screening effect in the ZnO nanostructure added to the fiber has been researched. Parangusan et al. studied cobalt-doped ZnO NPs as a filler in polyvinylidene fluoride hexafluoropropylene (PVDF-HFP) nanofibers, fabricated by electrospinning [94]. An increase in Co-doped ZnO nanofiller concentration enhanced the β-phase to 54.6% from 37.5% due to the interaction between the oppositely charged Co-ZnO surface and the –CF2-/-CH2-dipoles of PVDF-HFP. Addition of the Co-doped nanofiller increased the voltage output by more than 20 times compared to pure PVDF-HPF. In one study, PVDF and Y-doped ZnO NSs were mixed and electrospun to study the effect of adding Y-doped ZnO NSs with high d33 [47,95]. After adding the Y-doped ZnO NSs, the NSs led to knots in the PVDF fibers, which acted as stress concentrations and imparted better piezoelectricity on the fiber. The β-phase percentage for PVDF increased from 69% to 98% by adding the Y-ZnO because of the interfacial interaction of the filler and PVDF leading to more dipole orientation and the synergistic effect of a higher content of filler. The voltage output increased around three times.

Bairagi and Ali electrospun PVDF fibers with KNN/ZnO NR fillers and hence used three piezoelectric materials in their PENG [96]. The KNN and ZnO NRs acted as β-nucleating agents in the PVDF matrix during the electrospinning operation and the addition of the ZnO increased the power output 2.4 times compared to the PENG with only PVDF and KNN.

In one study, a breathable electrospun PVDF fiber with ZnO NRs grown on top of the PVDF fiber was developed [97]. The hydrothermal growth of ZnO NRs was limited to 60 °C and did not depolarize the PVDF. The open-circuit voltage increased ~2.3 times due to the ZnO NRs. The mat was measured to have the same breathability as cotton.

**Table 6:** Power, Voltage and Current Output for Electrospun Materials.

	PEMs	Form	Preparation Methods	V (V)	I (μA)	Resistor (MΩ)	Power Density (μW/cm^2^)	Force
[90]	PVDF/ZnO (27.3% by mass ZnO)	PVDF- Ø 219.4 nm ZnO- Ø 90 to 140 nm ~ 300 nm in length	Electrospinning + Dip Coating + Hydrothermal	1.12	1.6	0.7	0.2	140 Hz, 116 dB
[91]	PAN/ZnO	PAN Ø 650 ± 50 nm ZnO ~2.1 μm in length Ø 98 nm	Electrospinning + Heat Treatment + Hydrothermal	6.73	2.36	0.7	1.08	Impact force 8 N @ 2 Hz
[96]	PVDF/3% KNN/2%ZnO	ZnO Ø 79 nm PVDF Ø 300 nm	Electrospinning	25	1.81	10	11.31	Finger tapping
[92]	PVDF/ZnO NR (5% by wt.)	ZnO rods ~Ø 75 nm 830 nm long PVDF Ø 1.15 μm	Electrospinning	85 OC	2.2	-	-	Bending ~4 Hz
[94]	PVDF-HFP/Co-doped ZnO	Co-doped ZnO nanoparticles in PVDF-HFP	Hydrothermal + Electrospinning	2.8 OC	-	-	-	Tapping force of 2.5 N at 50 Hz
[95]	PVDF/Y-doped ZnO (15% by wt)	Y-ZnO NSs PVDF Ø 178 nm	Electrospinning	13	1.6	10	2	40 N and 0.8 Hz
[97]	PVDF/ZnO	ZnO NRs Length ~183 ± 153 nm Ø 30 ± 9 nm PVDF Ø 120 ± 100 nm	Electrospinning + Dip Coating + Hydrothermal	8.3	0.139	60	0.077	0.1 MPa 1 Hz
[93]	10 wt% ZnO NRs/P(VDF-TrFE)	ZnO NRs Length ~793 nm Ø 91 nm 0.98 μm	Electrospinning	61 OC	2.2 SC	-	-	Finger bending, 4 Hz

### 3.7. ZnO-Deposited Nanofilms

**Figure 8:**
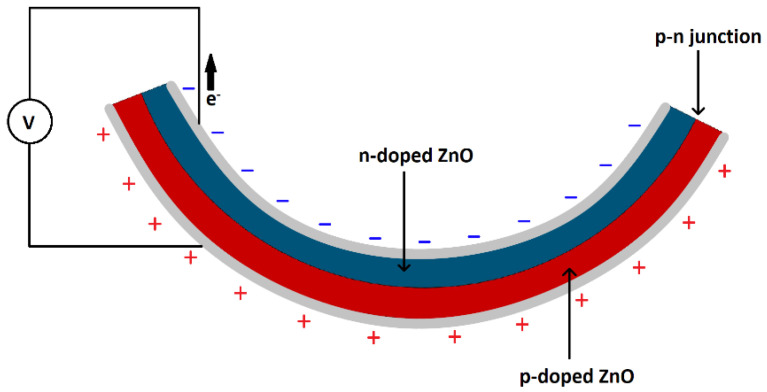
Side view of a nanofilm, comprising a P-doped region in red and an N-doped region in blue, being bent. The p-n junction helps reduce the screening effect and increases piezoelectricity.

ZnO-deposited nanofilms are ZnO films with a thickness of less than 1 µm that are created by a deposition process such as sputtering as shown in Figure 8. In general, there are less studies on ZnO thin films in the nanometer thickness range due to their low surface area as well as low piezoelectric properties [98]. The output power, voltage, and current of nanofilms are given in Table 7.

Several studies focus on decreasing the screening effect in these films. Kwon et al. synthesized a ZnO p–n homojunction thin film, composed of n-ZnO and phosphorus-doped p-type ZnO with PDMS on top to act as an insulation layer [99]. The p-n junction and insulation layer in the PENG reduced the screening effect and the PENG had an output power approximately two orders of magnitude higher than that of a pristine PENG. A consistent output voltage and current were measured over 5000 cycles without signal degradation. Shin et al. formed a CuO–ZnO heterostructure in the form of a film [100]. CuO (p-type) was sputtered onto PET followed by ZnO which formed a p–n junction and reduced the screening effect. The PET substrate worked as an insulator, reducing the screening effect as more excess electrons in the ZnO were being consumed by recombination. A seven-fold higher output voltage and an approximately one order of magnitude higher current density was obtained. In one study, a p-type Poly(3-hexylthiophene) (P3HT) polymer mixed with phenyl-C61-butyric acid methyl ester (PCBM) to improve carrier transport was placed on a ZnO thin film to form a p-n junction [101]. Holes greatly reduced the screening effect as they led to the combination of electrons. MoO_3_ was placed as an insulator underneath the electrode to reduce the screening effect. The ZnO/P3HT: PCBM-assembled piezoelectric power generator demonstrated an 18-fold enhancement in the output voltage and tripled the current. 

Porosity in these films can increase power output. Lee et al. developed a porous ZnO structure by annealing (at 850 °C) a sputtered ZnO thin film which led to minute pores emerging at the ZnO/Si (substrate) interface due to the ZnO bond breaking via thermal decomposition. The pores lead to an increase in d33 due to pore-induced bonding stain and the formation of stress concentrations. More than 90% of the pores were created approximately 100 nm away from the interface. By introducing a porous structure, the open output voltage of PENGs was enhanced by up to 7.5 times [102]. In one study, a nano-branched porous ZnO thin film composed by a network of randomly oriented wurtzite crystallites was formed [98]. Zn was sputtered to form a Zn film, after which the film was heated at 380 °C for 2 h, forming the porous ZnO structure. The more defective structure of the nano-branched ZnO thin films reduced free carrier mobility and the reduction in screening effect led to a ~3.75-times larger output voltage than nonporous ZnO films; however, the porous film was more fragile mechanically.

In one study, the effect of the thickness of ZnO thin films on piezoelectricity was studied [31]. Thicker films had a higher crystallinity and a preferential orientation along the c-axis direction, as well as a lower density of grain boundaries and larger crystal sizes. The highest generated output voltage (0.746 V) belonged to the thickest films on hard and flexible substrates, respectively. The underlying metal substrate had a strong influence on the morphology of the ZnO thin film surface, however it was more pronounced for the thinner samples and then was no longer visible for the thicker samples.

In one study, a ZnO thin film was deposited onto a Si substrate and had good c-axis orientation as well as a d33 of 49.7 pm/V [103]. Promsawat et al. deposited a ZnO thin film on a PET substrate via sputtering [104]. A strong crystallinity, a preferred orientation of crystal planes in the c-axis, as well as a uniform grain size were observed. The PENG produced an output of 14 µW when attached to a cantilever under a tip displacement of 5 mm.

**Table 7:** Power, Voltage and Current Output for Deposited Films.

	PEMs	Form	Preparation Methods	V (V)	I (μA)	Resistor (MΩ)	Power Density (μW/cm^2^)	Force
[99]	P doped ZnO	n-ZnO 200 nm p-ZnO 300 nm	Sputtering	~24	~6	10	-	Compression (0.5 MPa)
[100]	ZnO	n-ZnO 60 nm p-CuO 300 nm	Sputtering	~7.5 OC	~4.5 /cm^2^ SC	-	-	Compression 0.6 kg/cm^2^
[101]	ZnO	n-ZnO P3HT:PCBM	-	1.45	6.05/cm^2^	-	18% conversion efficiency	Bending 0.068% strain
[102]	ZnO	ZnO 68 nm pores	Sputtering + Annealing	0.003 OC	-	-	-	Compression 1.3 Hz 25 kPa
[98]	ZnO	ZnO Porous	Sputtering + Thermal Oxidation	2.2 V	-	-	-	Compression 25 Hz 20 N
[31]	ZnO	ZnO 1380 nm	Sputtering	0.746	-	-	-	Compression 30 N
[103]	ZnO	ZnO 800 nm	Pulsed laser deposition	0.095	0.000725	130	-	Striking with 0.4 kg/cm^2^ at 1000 Hz
[104]	ZnO	ZnO ~1 μm	Sputtering	2	150	0.750	18.7	Cantilever with tip displacement of 5 mm

## 4. Discussion and Conclusions

In this article, the suitability of ZnO as a lead-free piezoelectric material for use in PENGs was reviewed. Batra et al. synthesized Nd-doped ZnO NR with a d33 value of 512 pm/V, which is comparable to PZT in bulk form and thus ZnO is a good choice of piezoelectric material for PENG manufacturing [48]. 

Different types of ZnO PENG structures were reviewed from a power output maximization point of view. It is difficult to compare the different ZnO PENG structures in terms of power as different studies tested their PENGs under different loading conditions. However, comparing Table 3, Table 4, Table 5, Table 6 and Table 7, 1-3 composites have the highest power output of the studies reviewed. The high power output is due to the vertical alignment of the rods, high crystallinity along the c-axis, the use of methods to reduce the screening effect, and an incorporation of other piezoelectric materials. The 1-3 composites with NWs have high aspect ratios in the nanostructure, while 1-3 composite with NSs have higher stability and some studies have shown that they can produce even higher power output than NWs [59,77]. The 0-3 composites are easier to manufacture but there is lack of alignment between the ZnO nanostructures and hence they have lower power output. Deposited films have low power outputs and piezoelectricity and are typically manufactured via sputtering, which is an expensive process. In terms of electrospinning, ZnO is mostly used as a filler and increases the piezoelectricity of other piezoelectric materials.

## 5. Future Directions

Future directions of study are as follows:

1. Development of PENGs with multiple piezoelectric materials working synergistically together to increase power density. Further deconvolution of the interaction of different piezoelectric materials at the nanoscale is needed.
2. Development of PENGs that use multiple power output increasing techniques, such as doping, interfacial modification, surface passivation, addition of porosity, increasing thickness, annealing, poling, and the integration of multiple nanostructures (1D, 2D, and so on), which work together synergistically to increase power density and durability.
3. Frequency response characterization and modification. As most vibrations found in ambient conditions are of low frequency (<200 Hz [105,106,107]), the resonance frequency of the PENGs/PENGs attached to a vibrating body should be studied and tailored to be within this regime.
4. The complete characterization of piezoelectric properties of ZnO nanostructures so the properties can be used in finite element models to optimize PENG structures.
5. The use of PENGs in shear mode energy harvesters as typically the shear mode of a piezoceramic material offers improved energy harvesting potential compared to other modes [108].
6. ZnO PENG piezoelectric properties should be studied as a function of temperature if they are meant to be used at temperatures higher than room temperature. As the temperature of the PENG changes, its piezoelectric properties and impedance will change as well [109,110]. As a result, the optimal resistance value for maximum power transfer will change [109,110].

## Figures and Tables

**Table 1:** d33 Values for Pure ZnO Structures.

	d33 (pm/V)	Dimensions	Measurement Method	
**ZnO bulk**	12.4			[14]
**Nanowire**	0.4–9.5	Φ 150–500 nm L = 400–600 nm	AFM/PFM	[38]
	~9.2	Φ 150 ± 55 nm L = 2300 ± 120 nm	Nano Indenter	[39]
	7.5 ± 0.6	Φ 300 nm L = 2 µm	AFM/PFM	[40]
	11.8	Φ 150 nm L = 1.5 µm	AFM/PFM	[41]
	49.7	Φ 20 nm L = 500 nm	AFM/PFM	[34]
**Nanobelt**	14.3–26.7	65 nm thick 360 nm wide Tens of µm in length	AFM	[37]
**NSs**	6.48–18.9	1.5–4.5 nm thick Triangular side length ~8 μm	AFM	[42]
	80 ± 0.8	1.1 nm thick Laterally several mm	AFM	[33]
**Film**	5–5.3	Thickness 285–710 nm	AFM/PFM	[31]
